# Chromosomal abnormalities and copy number variations in fetal ventricular septal defects

**DOI:** 10.1186/s13039-018-0408-y

**Published:** 2018-11-28

**Authors:** Meiying Cai, Hailong Huang, Linjuan Su, Na Lin, Xiaoqing Wu, Xiaorui Xie, Gang An, Ying Li, Yuan Lin, Liangpu Xu, Hua Cao

**Affiliations:** Department of the Prenatal Diagnosis Center, Fujian Provincial Maternity and Children’s Hospital, affiliated hospital of Fujian Medical University, Fujian Key Laboratory for Prenatal Diagnosis and Birth Defect, Fuzhou, China

**Keywords:** Ventricular septal defects, Chromosomal microarray analysis, Chromosomal abnormality, Prenatal diagnosis

## Abstract

**Background:**

This study aimed to evaluate the applicability of chromosomal microarray analysis (CMA), rather than traditional chromosome analysis, in prenatal diagnosis of ventricular septal defects (VSDs) for superior prenatal genetic counseling and to reveal a potential correlation between submicroscopic chromosomal aberrations and VSDs.

**Results:**

Among the 151 VSD cases, 79 (52.3%) had isolated defects and 72 (47.7%) had additional ultrasound anomalies. Karyotype analysis identified 16 chromosomal abnormalities. Besides the 14 cases of chromosome abnormalities consistent with karyotype analysis, CMA identified an additional 20 cases (13.2%) of abnormal copy number variations (CNVs), of which 13 were pathogenetic CNVs, 5 were variations of uncertain clinical significance (VOUS) and 2 were benign CNVs. The detection rate of pathogenic CNVs in non-isolated-VSDs was significantly higher than that in isolated-VSDs (36.1% (26/72) vs. 1.3% (1/79), *p* = 0.001). We also found that CMA results indicating pathogenic abnormalities affected the rate of pregnancy termination.

**Conclusions:**

This study showed that CMA combined with cytogenetic analysis is particularly effective in identifying CNVs in fetuses with VSDs and can have an effect on obstetrical outcomes. The elucidation of the etiology of VSDs suggested that gene mutations or other factors may be implicated.

## Background

Congenital heart disease (CHDs) is one of the most common types of birth defects, affecting up to 8 in every 1000 babies born in the China [1]. Ventricular septal defects (VSDs) are the most common cardiac defects seen postnatally, accounting for 30–35% of all CHDs detected after birth and 10% of all fetal cases [2, 3]. Recent studies have shown that approximately 20–40% of VSDs are attributable to chromosomal aneuploidies or Mendelian diseases, while the remaining are attributable to non-Mendelian causes that are poorly understood [4–7]. Prenatal ultrasonography suggested that 33–47% percent of fetuses with VSD had chromosomal abnormalities, among which trisomy 18, trisomy 21, and DiGeorge syndrome were the most common [5, 8].

Genomic microarrays include micro-array based comparative genomic hybridization (aCGH) and single nucleotide polymorphism (SNP) arrays, both of which can detect micro-deletions and micro-duplications in the genome. Aside from the detection of CNVs, SNP arrays can detect uniparental disomy and chimeric DNA. In this study, we performed whole-genome scanning using SNP arrays as well as karyotyping of 151 fetuses that were diagnosed as having VSD by ultrasonic cardiography. We tried to elucidate genetic factors that cause fetal VSD and explore the clinical value of the application of CMA in the diagnosis of VSD in fetuses.

## Methods

### Patient data

We conducted a retrospective study on VSD cases diagnosed prenatally with ultrasound in the Prenatal Diagnosis Center of the Fujian Provincial Maternal and Children Health Hospital, from January 2017 to April 2018. Fetal samples were collected by amniocentesis (*n* = 85) or cord blood sampling (*n* = 66) depending on the gestation period. Amniotic fluid was collected by amniocentesis at the gestational age of 16–24 weeks and fetal blood was collected by cord blood sampling at a gestational age over 24 weeks. The fetuses underwent a routine ultrasonic scan, and fetal biometry was performed at a median gestational age of 25 + 5 (range, 18 + 3 to 33 + 4) weeks. This study was approved by the ethics committee at the Fujian Provincial Maternal and Child Health Hospital, and informed consent was obtained from the parents for invasive prenatal diagnosis. Once fetal pathogenic CNVs were confirmed, approximately 2.0 mL of peripheral venous blood was collected from their parents. DNA was extracted using the Gentra Puregene blood kit (QIAGEN, Santa Clara, CA, USA).

The cases were classified as isolated or non-isolated-VSD, the latter comprising cases with other cardiac anomalies, extracardiac structural anomalies, or sonographic soft markers. A total of 151 VSD cases were included, of which 79 (52.3%) cases were isolated-VSD and 72 (47.7%) cases were non-isolated-VSD (detailed information in Table 1).

**Table 1:** Phenotypic characteristics of 151 VSD fetuses

Classification	Number of fetuses	Number of Karyotype abnormality	Number of CHA		
			Pathogenic CNVs	VOUS	Benign CNVs
Isolated- VSD	79	1	1	1	1
Non-isolated-VSD	72	15	12	4	1
Total	151	16	13	5	2

*CMA* chromosomal microarray analysis, *CNVs* copy number variations, *VOUS* variation of uncertain clinical significance, *VSD* ventricular septal defect

### Cytogenetic analysis

Cultured amniocytes or lymphocytes were analyzed by regular karyotype analysis using the Giemsa banding technique at a resolution of 450–550 bands.

### CMA

Genomic DNA was directly extracted from uncultured amniotic fluid and cord blood samples using the QIAamp DNA Blood Mini kits (Qiagen, Germany). DNA was quantified using a NanoDrop spectrophotometer (Thermo Fisher Scientific, Waltham, MA, USA), according to the manufacturer’s instructions. The DNA quality was assessed by agarose gel electrophoresis. The genome-wide high-resolution SNP array CytoScan HD (Affymetrix Genome CytoScan 750 K, Affymetrix, Santa Clara, CA, USA) including both SNPs and oligonucleotide probes was used in this study. DNA (250 ng) was amplified, labeled, and hybridized according to the manufacturer’s protocol. Procedures for DNA digestion, ligation, polymerase chain reaction (PCR), fragmentation, labeling, and hybridization to the arrays were performed according to the manufacturer’s protocols. The CNV reporting filter was set at > 100 kb in size with a minimum set of 50 marker counts. The results obtained from the CytoScan arrays were analyzed with Chromosome Analysis Suite software (Affymetrix), using annotations of the genome version GRCH37 (hg19). The detected copy number gains or losses were systematically evaluated by comparing them with values available in the literature and the publicly available databases, including database of genomic variants (DGV, http://projects.tcag.ca/variation/), DECIPHER database (http://decipher.sanger.ac.uk/), the International Standards for Cytogenomic Arrays (ISCA, https://www.iscaconsortium.org/), and Online Mendelian Inheritance in Man (OMIM, http://www.omim.org). The CNVs were classified as benign, pathogenic, or variants of uncertain significance (VOUS) according to the American College of Medical Genetics (ACMG) guidelines [9]. Microarray analysis of DNA from maternal and paternal blood samples was used to determine whether CNVs detected in the fetal samples were inherited or de novo. All de novo CNVs were experimentally validated by fluorescence in situ hybridization (FISH).

### Statistical analysis

IBM SPSS Statistics 20 (IBM, Armonk, NY, USA) was used for statistical analysis. The detection rate of pathogenic variants in the isolated and non-isolated-VSD fetuses was compared between cytogenetic analysis and CMA. A value of *p* < 0.05 was considered statistically significant.

## Results

### Cytogenetic analysis of VSD fetuses

In the 151 VSD cases analyzed, karyotyping identified 16 chromosomal abnormalities. Among the 79 isolated-VSD fetuses, karyotyping identified only one chromosomal abnormality associated with 13p+, which was not found with CMA. However, of the 72 non-isolated-VSD fetuses, karyotyping identified 15 clinically significant chromosomal abnormalities, involving trisomy 18 (*n* = 5), trisomy 21 (*n* = 2), trisomy 13 (*n* = 1), Klinefelter syndrome (*n* = 1), deletions of 4q25q28 (*n* = 1), and duplications of 16p13.3 (*n* = 1), 22q11.2 (*n* = 1), and 8q21 (*n* = 1), and an unusual partial aneuploidy- monosomy 18 and trisomy 18 with one chromosome exhibiting a 18p11.32p11.31 microdeletion and 18p11.31p11.21 duplication. Additionally, we found one pericentric (9)(p12q13) inversion, which was not found with CMA (Table 2).

**Table 2:** Abnormal karyotyping results of VSD fetuses

Case	Karyotype	CMA results	Prenatal ultrasound	Postnatal outcome
1	47,XX,+ 18	arr[hg19](18) × 3	VSD; Posterior fossa widening; Single umbilical artery	TP
2	47,XX,+ 18	arr[hg19](18) × 3	VSD; TS; FGR	TP
3	47,XX,+ 18	arr[hg19](18) × 3	VSD; ARA; TS; Absence of nasal bone	TP
4	47,XY,+ 18	arr[hg19](18) × 3	VSD; Single umbilical artery; FGR	TP
5	47,XY,+ 18	arr[hg19](18) × 3	VSD; FGR; Absence of nasal bone; Overriding fingers	TP
6	47,XY,+ 21	arr[hg19](21) × 3	VSD; ARA; PVS; Absence of nasal bone	TP
7	47,XY,+ 21	arr[hg19](21) × 3	VSD; TGA	TP
8	47,XY,+ 13	arr[hg19](13) × 3	VSD; COA; LSVC; Single umbilical artery	TP
9	47,XXY	arr[hg19](1–22) × 2,(XXY) × 1	VSD; FGR	TP
10	46,XX,-18,+mar	arr[hg19]18p11.32p11.31(136,227-3,348,254) × 1,18p11.31p11.21(3,350,736-13,083,388) × 3	VSD; COA; PVS; AOO	TP
11	46,XY,del,(4)(q25q28)	arr[hg19]4q25q28.1(112,192,577-127,874,789) × 1	VSD; widening of left lateral ventricle	TP
12	46,XY,add(16)(p13.3)	arr[hg19]16P13.3(85,880-536,631) × 1,17q24.2q25.3(64,966,574-81,041,823) × 3	VSD; LSVC; widening of left lateral ventricle	TP
13	47,XY,add(22)(q11.1)	arr[hg19]22q11.1q11.21(16,888,899-18,649,190) × 4	VSD; HA; Single umbilical artery	TP
14	46,XX,add(8)(q21)	arr[hg19]8q21.2q23.3(86,553,128-114,877,447) × 3	VSD; PTA	TP
15	46,XX,inv.(9)(p12q13)	arr[hg19](1–22) × 2,(XX) × 1	VSD; choroid plexus cysts	TD
16	46,XY,13p+	arr[hg19](1–22) × 2,(XY) × 1	VSD	TD

*AOO* augmentation of oval, *ARA* aortic ride across, *COA* coarctation of the aorta, *CMA* chromosomal microarray analysis, *FGR* fetal growth restriction, *HA* hypoplastic aorta, *HLHS* hypoplastic left heart syndrome, *LSVC* left superior vena cava, *PTA* persistent truncus arteriosis, *PVS* pulmonary valve stenosis, *SV* single ventricle, *TD* term delivery, *TGA* transposition of the great arteries, *TP* termination of pregnancy, *TS* tricuspid stenosis, *VSD* ventricular septal defect

### Detection rates of CMA with karyotyping

CMA was performed in 151 VSD fetuses, with 22.5% (34/151) of the cases showing chromosomal abnormalities. Fourteen of these cases were also identified with the karyotype analysis, but the other 20 were identified only with CMA, of which 13 were pathogenetic CNVs, 5 were VOUS, and 2 were benign CNVs. Among the 13 fetuses with pathogenic CNVs, only one fetus had isolated-VSD. These 13 CNVs involved deletions of 3q24q25.1, 15q24.1q24.2, and 22q11.21; duplications of 3q29, 7q11.23, 17p11.2, 22q11.21, 22q11.1q11.21, and Xp28; and loss of heterozygosity of 16q23.2q24.3 and 16p13.3p12.3. There were 7 cases with pathogenic CNVs related to known chromosomal disorder syndromes- DiGeorge syndrome (*n* = 4), Schmid-Fraccaro syndrome (*n* = 2), and Potocki-Lupski syndrome (*n* = 1) (Table 3).

**Table 3:** Chromosomal microarray analysis of VSD fetuses with normal karyotyping results

Case	CMA results	Size (Mb)	Prenatal ultrasound	Pathogenicity classification	Obstetrical outcomes	Inheritance
1	arr[hg19]22q11.21(18,648,855-21,800,471) × 1	3.1	VSD; RAA; vascular circle; ALSA	P (DiGeorge syndrome)	TP	de novo
2	arr[hg19]22q11.21(18,648,855-21,800,471) × 1	3.1	VSD; ARA; PA; thymic hypoplasia	P (DiGeorge syndrome)	TP	de novo
3	arr[hg19]22q11.21(18,631,364-20,729,389) × 1	2.0	VSD; thymic hypoplasia	P (DiGeorge syndrome)	TP	de novo
4	arr[hg19]22q11.21(18,648,855-21,800,471) × 1	3.1	VSD; RAA	P (DiGeorge syndrome)	TP	de novo
5	arr[hg19]22q11.21(18,648,855-21,800,471) × 1	3.1	VSD; ARSA; TR; LSVC; Single umbilical artery	P (Cat Eye Syndrome)	TP	de novo
6	arr[hg19]22q11.1q11.21(16,888,899-18,649,190) × 4	1.7	VSD; ARSA; TR; LSVC; Single umbilical artery	P (Cat Eye Syndrome)	TP	de novo
7	arr[hg19]17p11.2(16,567,623-18,743,354) × 3	2.1	VSD; PA; FGR	P (Potocki-Lupski Syndrome)	TP	de novo
8	arr[hg19]Xp28(152,713,658-153,421,838) × 3	0.69	VSD; TR	P	TP	de novo
9	arr[hg19]3q24q25.1(143,476,996-151,222,561) × 1	7.7	VSD; ocular hypertelorism; widening of lateral ventricle	P	TP	de novo
10	arr[hg19]15q24.1q24.2(72,965,465-75,567,135) × 1	2.6	VSD; PA; FGR; Absence of nasal bone	P	TP	de novo
11	arr[hg19]3q29(195,743,957-197,386,180) × 3	1.6	VSD	P	TP	de novo
12	arr[hg19]7q11.23(72,701,098-74,069,645) × 3	1.3	VSD; Left kidney dysplasia	P	TP	de novo
13	arr[hg19]16q23.2q24.3(79,800,878-90,146,366)hmz,16p13.3p12.3(94,807-19,302,326)hmz	10.3	VSD; PVS; Left kidney dysplasia; FGR	P	TP	UPD
14	arr[hg19]11P15.1P14.3(20,745,930-21,780,075) × 3	1.0	VSD; Right hydronephrosis; widening of left lateral ventricle	VOUS	TP	De novo
15	arr[hg19]4q24(106,284,925-107,545,257) × 3	1.2	VSD; ARSA; FGR	VOUS	TD	De novo
16	arr[hg19]9q21.33q22.1(89,868,507-90,975,015) × 3	1.1	VSD	VOUS	TD	De novo
17	arr[hg19]16p13.11(14,897,401-16,534,031) × 1	0.5	VSD; Left ventricular hyperechoic	VOUS	TD	De novo
18	arr[hg19]2q31.2q31.3(180,558,684-181,901,189) × 3	0.5	VSD; OFB	VOUS	TD	De novo
19	arr[hg19]5q14.1(76,983,283-77,512,158) × 3	0.5	VSD	B	TD	Maternal
20	arr[hg19]10q21.1(59,095,330-60,684,488) × 1	1.5	VSD; TR	B	TD	Maternal

*ALSA* aberrant left subclavian artery, *ARA* aortic ride across, *ARSA* aberrant right subclavian artery, *B* benign, *CMA* chromosomal microarray analysis, *CNVs* copy number variations, *FGR* fetal growth restriction, *LSVC* left superior vena cava, *OFB* oval flaps bulging, *P* Pathogenic, *PA* pulmonary atresia, *PVS* pulmonary valve stenosis, *RAA* right aortic arch, *TR* tricuspid regurgitation, *TD* term delivery, *TP* termination of pregnancy, *UPD* uniparental disomy, *VOUS* variation of uncertain clinical significance, *VSD* ventricular septal defect, *VC* vascular circle

### Comparison of pathogenic CNV detection rates

Overall, the detection rate of pathogenic CNVs with CMA was significantly higher than that with karyotype analysis (17.9% vs. 10.6%, *p* < 0.05). The detection rate of CNVs in non-isolated-VSDs was significantly higher than that in isolated-VSDs (36.1% (26/72) vs. 1.3% (1/79), *p* = 0.001).

### Inheritance analysis and obstetrical outcomes

We screened the hereditary information of 27 families in which karyotyping showed chromosomal abnormalities (9 cases of chromosome aneuploidies were exempted) and abnormal CNVs. Parental analysis showed that that in 5 fetuses the abnormalities were inherited from unaffected parents (Table 4), and in 18 cases, the CNVs occurred de novo. The reasons for termination of pregnancies in the 28 women analyzed were chromosome abnormalities (*n* = 14), pathogenic CNVs (*n* = 13), and VOUS CNVs (*n* = 1). We also found that in 2.5% (2/79) of isolated-VSD cases and 44.4% (32/72) of non-isolated-VSD cases, pregnancy was terminated.

**Table 4:** Chromosomal abnormalities inherited from unaffected parents in VSD fetuses

Case	Prenatal ultrasound	Abnormal fetal chromosomes	Pathogenicity classification	Inheritance	Postnatal outcome
1	VSD	46,XX,inv.(9)(p12q13)	B	Maternal	TD
2	VSD	46,XY,13pstk+	B	Maternal	TD
3	VSD, TR	arr[hg19]5q14.1(76,983,283-77,512,158) × 3	B	Maternal	TD
4	VSD, single umbilical artery	arr[hg19]10q21.1(59,095,330-60,684,488) × 1	B	Maternal	TD
5	VSD, RAA, left kidney dysplasia	arr[hg19]16q23.2q24.3(79,800,878-90,146,366)hmz,16p13.3p12.3(94,807-19,302,326)hmz	P (LOH)	Maternal Normal phenotype	TP

*B* benign, *P* pathogenic, *RAA* right aortic arch, *TD* term delivery, *TP* termination of pregnancy, *TR* tricuspid regurgitation, *VSD* ventricular septal defect, *LOH* loss of heterozygosity

## Discussion

The development of CMA technology, including aCGH and SNP arrays, in the early 2000s provided a new research tool for chromosomal analysis. Since then, a number of studies have used CMA to locate novel candidate genes involved in heterotaxy [10], isolated tetralogy of Fallot (TOF) [11], and left-sided CHD [12], as well as novel genomic regions of interest [13]. However, little is known about the involvement of CNVs in either isolated or non-isolated-VSDs. Therefore, we used CMA combined with cytogenetic analysis to identify chromosomal abnormalities and CNVs in fetuses with VSDs, for superior prenatal genetic counseling and revealed a potential correlation between submicroscopic chromosomal aberrations and VSDs.

VSDs are attributable to both Mendelian diseases or chromosomal aneuploidies, as well as to non-Mendelian causes [2]. In our study, karyotype analysis identified 16 chromosomal abnormalities in 151 VSD cases. Among the isolated-VSD fetuses, karyotyping only identified one chromosomal abnormality associated with 13p+, a type of heterochromatin polymorphism, which was not found with CMA. Heterochromatin is regarded as a morphological manifestation of genetic silencing [14]. However, in the non-isolated-VSD fetuses, karyotyping identified clinically significant chromosomal abnormalities involving trisomy 18, trisomy 21, trisomy 13, Klinefelter syndrome, 22q11.2 deletion, and trisomy 18 with one chromosome exhibiting a 18p11.32p11.31 microdeletion and 18p11.31p11.21 duplication. A previous study has reported the association between VSD and major aneuploidies (trisomy 21, trisomy 18, trisomy 13, and Klinefelter syndrome), as well as 22q11.2 deletion syndrome [15], confirming our findings. Karyotype analysis also identified deletion of 4q25q28, and duplications of 16p13.3 and 8q21. These three chromosomal abnormalities are associated with abnormal cardiac development, according to the DECIPHER database. Additionally, we found one pericentric (9)(p12q13) inversion, which was not found with CMA. Some reports have predicted that the genes responsible for normal heart development could be present on chromosome 9, around the p11-q13 region, which might be rendered defective during the process of inversion and thereby resulted in CHD; however, this theory needs further study.

According to the study [16], CMA is unable to replace karyotype analysis due to its failure in detecting chromosome translocations or inversions. But CMA is superior to karyotype analysis because of its high accuracy and resolution in detecting and identifying CNVs, without the need for amniotic fluid cell culture. We observed that the detection rate of pathogenic abnormalities with CMA was significantly higher than with karyotype analysis. According to some reported studies, the yield of CMA in prenatal evaluation ranges from 6.6 to 19.2% [17–20]. The actual rates of clinical detection in our cohort are consistent with the reported studies.

In this study, the most frequently detected CNVs in VSD cases were 22q11.2 deletions. CHDs are known to be associated with 22q11.2 microdeletion [21]. According to the DECIPHER database, *TBX1* is the key causative gene for CHD phenotypes resulting from the 22q11.2 deletion. Besides 22q11.2 deletions, we identified two CNVs associated with Cat Eye Syndrome [22] and Potocki-Lupski Syndrome [23]. Patients with these syndromes display a range of physical and mental disabilities as well as CHDs, including VSDs. Although there is no reported correlation between Xq28 duplication and VSDs, this fragment is important for intellectual development and has been confirmed as a pathogenic CNV [24]. It is well accepted that when VSD is detected in the presence of other structural anomalies, it is more likely related to genetic disorders [25]. We identified a diverse set of four CNVs associated with rare chromosomal syndromes- 3q24q25.1 and 15q24.1q24.2 and the two microduplications, 3q29 and 7q11.23. These syndromes display a range of physical and mental disabilities as well as congenital organ malformations, including VSD. Syndromic VSDs are related to a variety of etiologies such as microscopic and sub-microscopic chromosomal abnormalities, monogenic syndromes, as well as epigenetic and environmental factors [26]. We also found one VSD case with loss of heterozygosity in 16q23.2q24.3 and 16p13.3p12.3. CMA on the parents showed that it was a result of maternal uniparental disomy, which was a classified pathogenic variation.

We observed five cases of VOUS. The result is consistent with the frequency reported in other studies using similar CMA [6, 18]. One case had a 1.0 Mb duplication in chromosome 11p15.1p14.3 that involved an OMIM gene, NELL1, which is associated with the differentiation of brain cells and the growth, differentiation, and mineralization of osteoblasts. The parents’ CMA results were normal. Thus, the fetal CNV was de novo, but its clinical significance is uncertain. Due to the non-isolated-VSDs with other severe ultrasound anomalies, the parents opted to terminate the pregnancy.We checked the reference genes involved in the other four cases of VOUS, and none of them are known to be associated with embryonic heart development.The parents opted to delivery the pregnancy. As the use of CMA for genetic disorders expands, increasing data on CNVs is expected to decrease the number of VOUS remarkably in the future.

In this study, the detection rate of CNVs in non-isolated-VSDs was significantly higher than that in isolated-VSDs. In contrast, An et al. [27] showed that there was no significant difference in the detection rate between isolated-VSD and not-isolated-VSD. One reason for the varying detection rates might be due to different prenatal ultrasound equipment and array probe scales. We found that 2.5% (2/79) of isolated-VSD cases and 44.4% (32/72) resulted with termination of pregnancy, with the reasons being chromosome abnormalities, pathogenic CNVs, and other severe ultrasound anomalies. We also found that pregnant women were more likely to continue pregnancy when known chromosomal abnormalities were excluded. This highlights the importance of improving genetic counseling, providing psychological support, and raising awareness of VSDs in parents to reduce unnecessary termination of pregnancies.

## Conclusion

The accuracy of CMA testing is significant in cases of prenatally detected CHD, for both isolated- and non-isolated-VSD cases. In conclusion, CMA combined with cytogenetic analysis is particularly effective in identifying chromosomal abnormalities and CNVs in fetuses with VSDs, which could aid parental counselling. Based on this study and others [28], a majority of the cases still remain elusive, suggesting that different gene mutations or other factors may be implicated in the etiology of VSDs.

## Abbreviations

CHD: Congenital heart disease
CMA: Chromosomal microarray analysis
CNV: Copy number variation
FISH: Fluorescence in situ hybridization
LOH: Loss of heterozygosity
PCR: Polymerase chain reaction
SNP: Single nucleotide polymorphism
VOUS: Variation of uncertain clinical significance
VSD: Ventricular septal defects
